# Comparison of Margin Quality for Intersegmental Plan Identification in Pulmonary Segmentectomy †

**DOI:** 10.3390/medicina61030535

**Published:** 2025-03-19

**Authors:** Selcuk Gurz, Yurdanur Sullu, Leman Tomak, Necmiye Gul Temel, Aysen Sengul

**Affiliations:** 1 Department of Thoracic Surgery, Ondokuz Mayis University, Samsun 55270, Turkey; aysentaslak@yahoo.com; 2 Department of Pathology, Ondokuz Mayis University, Samsun 55270, Turkey; yurdanursullu@yahoo.com; 3 Department of Biostatistics and Medical Informatics, Ondokuz Mayis University, Samsun 55270, Turkey; lemant@omu.edu.tr; 4 Department of Thoracic Surgery, Samsun University, Samsun 55080, Turkey; necmiyegultemel@gmail.com

**Keywords:** indocyanine green, inflation–deflation, intersegmental margin, pulmonary nodule, segmentectomy

## Abstract

*Background and Objectives:* Insufficient margin in lung cancer is associated with an increased locoregional recurrence rate. In pulmonary segmentectomy, two commonly used methods for identifying the intersegmental plane are inflation–deflation and indocyanine green dyeing. The aim of this study was to compare these two methods in terms of quality margins and to evaluate their superiority. *Materials and Methods:* A total of 63 patients who underwent segmentectomy via video-assisted thoracoscopic surgery (VATS) for pulmonary nodules and underwent preoperative planning with 3D modeling between October 2020 and February 2024 were included in this study. The location of the nodule and the distance to the intersegmental margins were virtually measured preoperatively using an open-source 3D modeling system. Patients were grouped according to the method of identifying the intersegmental margins. Group 1 included segmentectomies performed by the inflation–deflation method (n = 42), and Group 2 included segmentectomies performed by systemic indocyanine green (ICG) injection (n = 21). The area where the histopathological nodule was measured closest to the intersegmental margin was recorded. Values within (+/−10 mm) compared to the value measured in the three-dimensional model were considered successful. The obtained data were statistically compared between the groups. *Results:* There was no difference between the groups in terms of virtual and pathological margins. However, in terms of margin quality, the rate of deviation detected in the pathological margin compared to the measured virtual margin was significantly different between the groups (*p* = 0.04). Accordingly, the success rate was 64.3% in Group 1 and 90.5% in Group 2 (*p* = 0.05). In Group 1, the failure rate was highly against the adjacent parenchyma. There was no significant difference between the groups in the analysis of simple and complex segmentectomies. *Conclusions:* Intersegmental plane identification with indocyanine green increases the margin quality by defining resection margins closer to the virtual margins. In the inflation–deflation method, unnecessary parenchymal loss occurs due to disadvantages in identifying intersegmental margins.

## 1. Introduction

Segmentectomy is one of the most complex procedures among anatomical resections in lung surgery. It is the preferred type of resection in cases of benign pathologies and lung cancer patients with compromised pulmonary function. However, in recent years, the observation that early-stage lung malignancies treated with segmentectomy achieve survival outcomes comparable to lobectomy has increased the significance of this procedure. In particular, a study conducted by the Japan Clinical Oncology Group (JCOG) demonstrated that patients who underwent segmentectomy for semi-solid/solid nodules smaller than 2 cm had superior survival and pulmonary function outcomes compared to those who underwent lobectomy [1]. This study also emphasizes the importance of performing segmentectomy in accordance with oncological principles.

One of the most critical factors influencing survival in anatomical segmentectomy is the inclusion of segmental margins in the resection [2]. Matsuura stated that precise determination of the intersegmental plane for pulmonary segmentectomy is critical to preserving pulmonary function and preventing locoregional recurrence [3]. Nevertheless, the characteristics of ideal margins to avoid local recurrences after surgical resection have been defined by guidelines (a margin at least as large as the tumor diameter for lesions > 2 cm or 2 cm for tumors > 2 cm) [4]. Various techniques are used to determine these margins. Although the jet ventilation technique and electrocautery-assisted resection are recommended for identifying segmental boundaries, advancements in video-assisted thoracic surgery (VATS) have significantly increased the importance of performing segmentectomy through minimally invasive approaches. The use of automatic sealing and cutting devices (endostaplers) through smaller incisions has become more prominent in modern surgical practice [5]. The inflation–deflation method (IDM) is widely used to identify the intersegmental plane in VATS segmentectomies [6,7]. Moreover, in anatomical segmentectomy, following the division of the vascular structures of the segment to be resected, systemic injection of indocyanine green (ICG) under near-infrared fluorescence imaging has been increasingly adopted in recent years for identifying the intersegmental plane [8]. In their study comparing the two methods, Huang et al. showed that both methods clearly revealed the intersegmental plan, and the plans determined by the two methods were completely compatible [9]. However, Sun et al. reported that the ICG method was significantly faster than the IDM in clarifying the intersegmental plan and significantly shortened the operative time [10]. Although the advantages and disadvantages of these two methods have been well documented in comparative studies, no clear consensus has been reached regarding their superiority in precisely defining the true intersegmental plane [11,12]. In this study, we aimed to evaluate both methods by comparing virtual intersegmental margins determined preoperatively through three-dimensional (3D) modeling with histopathological margins.

## 2. Materials and Methods

### 2.1. Study Design, Data Source, and Patients

**Figure 1:**
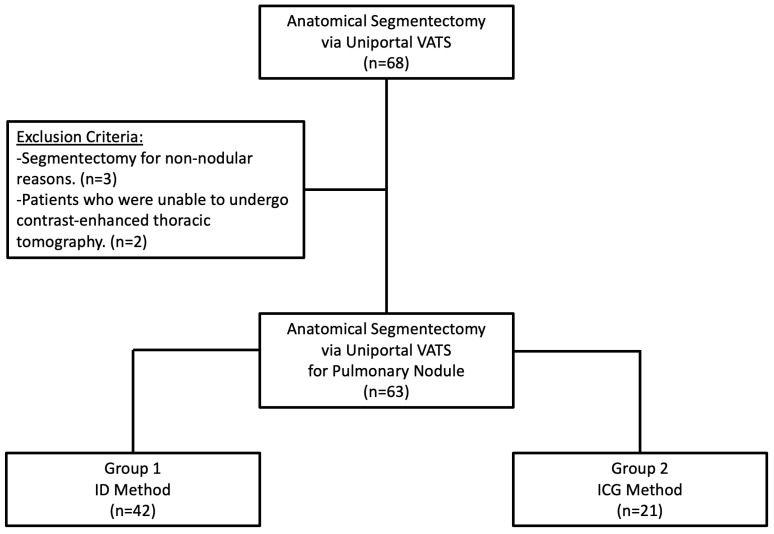
Flowchart of this study.

Between October 2020 and February 2024, data from 68 patients who underwent uniportal VATS segmentectomy were collected. Patients who were unable to undergo contrast-enhanced thoracic tomography and those who underwent segmentectomy for reasons other than nodules were excluded from this study. As a result, 63 patients who underwent VATS segmentectomy for pulmonary nodules were included in this study. A flowchart of this study is shown in Figure 1.

The selection criteria for uniportal VATS segmentectomy included (I) ground glass opacity (GGO), semi-solid, or solid nodules smaller than 2 cm; (II) lung cancer patients with inadequate pulmonary reserve for lobectomy; and (III) patients with pulmonary metastases or benign lesions unsuitable for wedge resection.

Patients were classified according to the method used for intersegmental plane identification. Group 1 included segmentectomies identified using the IDM (n = 42). Group 2 included segmentectomies identified using systemic ICG injection (n = 21).

All patients underwent preoperative 3D lung reconstruction. The shortest distance between the nodule and the intersegmental plane on the 3D model was measured as the “virtual margin” (VM). The shortest distance between the tumor and the stapler, measured histopathologically, was recorded as the “pathological margin” (PM). The difference between these two measurements was defined as the “Margin Deviation” (MD). Additionally, margin success was defined as an MD of less than 10 mm, while a deviation greater than 10 mm was considered a failure. A 10 mm threshold was established based on the thickness of the endostapler utilized for intersegmental plane division.

### 2.2. Three-Dimensional Reconstruction and Virtual Measurements

**Figure 2:**
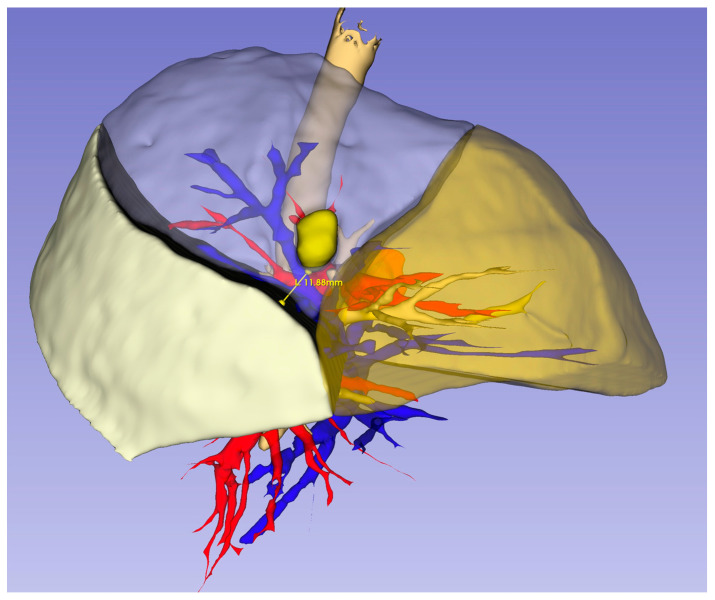
Virtual margin of the nodule to the intersegmental plane on preoperative 3D modeling.

The 3D reconstruction was performed as stated in our previous study [13]. All patients underwent preoperative CT imaging using a 64-detector MDCT scanner (GE Healthcare Discovery CT750 HD, Milwaukee, WI, USA) with iohexol (Opaxol) as the contrast agent. A total of 70 mL of non-ionic contrast medium (350 mg I/mL) was administered into the antecubital vein at a rate of 2 mL/s via a power injector. The acquired Digital Imaging and Communications in Medicine (DICOM) data were subsequently transferred to a dedicated workstation. Contrast-enhanced CT images were utilized to generate 3D models through a software-based analysis using the “Segment Editor” and “Segmentation” tools integrated into the open-source 3D Slicer software (version 5.1.1, https://www.slicer.org, accessed on 22 May 2023). To achieve optimal results, 3D modeling was conducted using contrast-enhanced CT images with a slice thickness of 1.25 mm or less. Pulmonary reconstruction was carried out by identifying bronchovascular structures, segmental divisions, and nodule localization. Properly timed contrast administration facilitated the automatic generation of a 3D model of the pulmonary arteries and veins. The intersegmental planes within the lobe containing the target segment were delineated based on venous anatomy, and the shortest distance between the nodule and the segmental demarcation was measured and documented (Figure 2). All reconstructions were conducted by the operating surgeon and independently assessed and confirmed by two radiology specialists.

### 2.3. Sugical Technique

**Figure 3:**
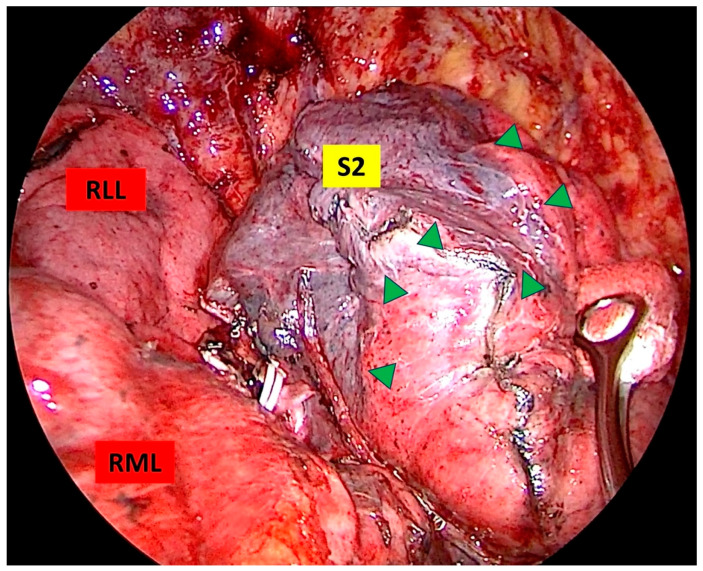
Determination of the intersegmental plane by the inflation–deflation method. The intersegmental plan is marked with green triangles (RLL: right lower lobe, RML: right middle lobe, S2: posterior segment of the upper lobe).

**Figure 4:**
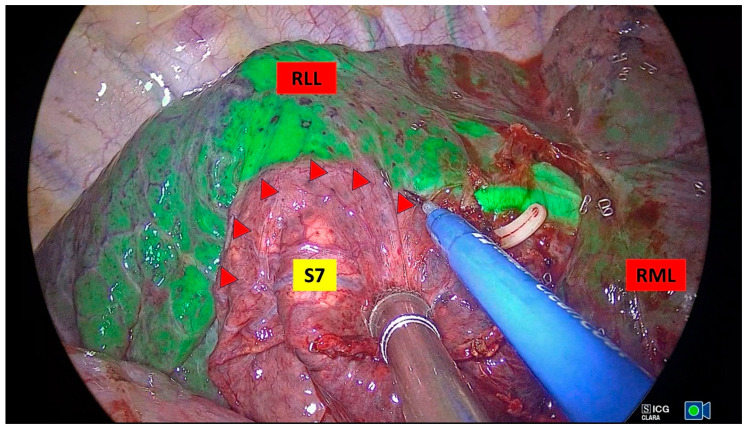
Determination of the intersegmental plane by systemic ICG injection under infrared fluorescence (Endovision System used IMAGE1 S™ Rubina^®^, Karl Storz, Tuttlingen, Germany). The intersegmental plan is marked with red triangles (RLL: right lower lobe, RML: right middle lobe, S7: mediobasal segment of the lower lobe).

The patient was positioned in lateral decubitus with the healthy side dependent. A double-lumen endotracheal tube was inserted, and general anesthesia with single-lung ventilation was administered. For uniportal surgery, a single 3–4 cm incision was made parallel to the intercostal space at the 5th or 6th intercostal level, between the anterior and midaxillary lines, and routinely protected with a silicone wound retractor. Visualization was achieved using a 10 mm 30° thoracoscope (HOPKINS^®^ Forward−Oblique 30° Telescope, Karl Storz, Tuttlingen, Germany), while an endoscopic sealing and dissecting device (LigaSure™ Maryland Jaw, Minneapolis, MN, USA) facilitated tissue division. The preferred instruments for vascular and bronchial dissection included a node grasper (Genings Snake), Dennis dissector clamp, Harken clamp, right clamp, and aspirator. To optimize lung manipulation, hilar dissection and routine lobe-specific lymph node dissection were performed in all cases. Segmental bronchi were transected using an endostapler, and vascular structures were transected using an endostapler or Hem-o-lock. Intersegmental planes were defined by two methods. In the IDM, the lung is inflated after dividing the segmental bronchus. Then, mechanical ventilation is stopped, observing that the segment to be resected remains markedly deflated. A distinct border is formed between the remaining inflated segments and the deflated segment (Figure 3). Systemic ICG injection with an infrared imaging system is performed by injecting the drug intravenously (0.15–0.25 mg/kg) after dividing the vascular structures of the target segment. Tissues that are reached by the drug appear green, and segments that are not reached by the drug remain normal in color. Thus, the intersegmental planes are clearly defined (Figure 4). The intersegmental planes were sealed using an endostapler to ensure anatomical separation. Although macroscopically safe surgical margins were maintained, all specimens were subjected to intraoperative frozen section analysis to histopathologically confirm negative resection margins and exclude lymph node metastases. Our segmentectomies were divided into simple segmentectomies and complex segmentectomies according to surgical procedures and intersegmental planes. Simple segmentectomies included right S6, left S6, left S1+2+3, and left S4+5 and were completed by creating only one linear intersegmental plane. The remaining segmentectomies were classified as complex segmentectomies and were completed by creating more than one intersegmental plane [14]. At the completion of the procedure, a 28Fr chest tube was inserted through the same incision, followed by the establishment of a negative pressure system with an underwater seal. Postoperative evaluation included routine chest X-ray imaging for all patients.

### 2.4. Statistical Analyses

Statistical analyses were performed with SPSS 21.0 for Windows. Data were presented as mean ± standard deviation (SD), as median (interquartile range: IQR), and as frequency (%). The Shapiro–Wilk test was used to analyze the normal distribution assumption of the quantitative outcomes. The data were analyzed by the Mann–Whitney test for non-normal data. The frequencies were compared using the Pearson Chi-square, Continuity Correction Chi-square, and Fisher Exact test. A *p* value less than 0.05 was considered statistically significant.

## 3. Results

A total of 63 patients who successfully underwent uniportal VATS segmentectomy were included in this study. The demographic characteristics and surgical outcomes of the patients are summarized in Table 1.

During segmentectomy, 42 patients in Group 1 underwent intersegmental plane identification using the IDM, while 21 patients in Group 2 underwent intravenous ICG injection for intersegmental plane identification. All nodules were resected via segmentectomy without a prior histopathological diagnosis, and the malignancy rate was 82.5%. The most common pathology was primary lung malignancy (73%), followed by secondary lung malignancies (9.5%). There was no statistically significant difference between the groups in terms of diagnosis.

No significant differences were observed between the groups regarding age, sex, nodule size, or follow-up duration. The most frequently performed segmentectomy type was complex segmentectomy in both groups (Group 1: 59.5%, Group 2: 81%), with no significant intergroup differences. The most commonly resected segments were S6 (22.2%) and S1 (20.6%). The intraoperative complication rate was 3.2%, and the postoperative complication rate was 9.5%, with no statistically significant difference between the groups. The intraoperative complications observed included minor, manageable bleeding in two patients, which did not require conversion to thoracotomy. The most common postoperative complication was prolonged air leak (n = 3), and one patient required redo VATS for this reason. The median chest tube removal time was 2 days (IQR: 1), and the median postoperative hospital stay was 3 days (IQR: 2).

The VMs of pulmonary nodules were measured (Group 1/Group 2: 21.14 ± 6.44 mm/22.28 ± 8.30 mm), and no significant difference was found between the groups. The PMs were measured in postoperative pathology specimens (Group 1/Group 2: 14.14 ± 9.23 mm/17.19 ± 9.87 mm), with no significant difference detected between the groups. The difference between the VM and PM was recorded as the MD (Group 1/Group 2: 8.28 ± 5.52 mm/5.19 ± 3.64 mm), and a statistically significant difference was observed between the groups (*p* = 0.040). The overall margin success rate was 73% for all patients. The margin success rate was 64.3% in Group 1 and 90.5% in Group 2, but the difference between the groups was not statistically significant (*p* = 0.057). A comparison of intersegmental margin measurements and margin quality between the groups is presented in Table 2.

## 4. Discussion

In pulmonary segmentectomy, the identification of intersegmental planes is one of the most critical steps in adhering to oncological principles. There is no definitive method to histopathologically confirm that a segment has been resected along the correct anatomical demarcations. However, the distance between the nodule and the surgical margin within the resected segment (surgical margin) is a key oncological criterion and one of the most influential factors affecting survival. Various techniques are used to define margin demarcations. Although the jet ventilation technique and electrocautery-assisted resection have been recommended for segmental demarcation identification, advancements in VATS techniques have increased the significance of performing segmentectomy through minimally invasive approaches using endostaplers via much smaller incisions [5,15]. The identification of intersegmental planes through ventilation is not always satisfactory. Therefore, in recent years, the systemic injection of ICG under near-infrared fluorescence imaging has been increasingly used for intersegmental plane identification following the ligation of vascular structures of the segment to be resected [16]. An effective and reliable ICG application depends on the accurate identification of the segment’s vascular structures.

In this study, the distance of the nodule from the segmental demarcations was virtually measured using preoperative 3D modeling. Additionally, the vascular anatomy identified through preoperative 3D modeling provided guidance during intraoperative dissection, enhancing the reliability of intravenous ICG application. The surgical margin, measured histopathologically, was compared with the virtual margin. The consistency in the two methods used for intraoperative intersegmental plane identification with preoperative 3D planning was evaluated. Since the study groups were formed based on the two different methods, the selection of the technique was random. Due to the limited availability of ICG in our country, intravenous ICG-based intersegmental plane identification was performed when the drug was accessible, whereas the inflation–deflation method was routinely used otherwise. Compared to the inflation–deflation method, intravenous ICG-based identification resulted in intersegmental planes that were closer to the virtual demarcations. The findings of this study suggest that ICG application provides a more reliable assessment of margin quality from an oncological perspective.

Studies comparing these two methods have demonstrated that intersegmental planes identified using ICG are wider and hold greater oncological value [11]. In this method, where the tissues reached by the drug appear green and segmental planes become more distinct, failure to accurately divide the vascular structures of the target segment may lead to inadequate resection margins or unnecessary expansion of the resection area. Insufficient margins can result in surgical outcomes that do not align with oncological principles [17]. An excessively wide margin is inconsistent with the principles of parenchyma-sparing surgery and the goal of preserving pulmonary function. Furthermore, the feasibility of the ICG method requires the installation of a costly technical infrastructure (such as an ICG-guided near-infrared fluorescence imaging system), the procurement of expensive ICG drugs, and a 3D modeling system to determine the correct vascular anatomy. Bedat et al. emphasized that ICG fluorescence imaging provides a faster and more accurate intersegmental plan determination but requires specialized equipment and may incur additional costs [18]. In our study, patient-specific vascular anatomy was identified using the 3D modeling method, guiding dissection and division. As a result, in 33.3% of the patients, intersegmental planes were delineated using ICG, ensuring its most effective application. In the remaining patients, the IDM was used to define segmental planes, while segmentectomy was performed with careful distal vascular dissection to preserve the vascular structures of adjacent segments.

Several conditions, such as emphysematous lung disease, extensive anthracosis, and drug allergies, have been identified, where each of these two methods has its own advantages and disadvantages [12]. The broncho-alveolar system of the lung contains microscopic structures, such as Kohn’s pores and Lambert’s canals, which facilitate collateral pathways between adjacent alveoli and bronchioles [4,19]. In the IDM, the presence of collateral airways may complicate the identification of intersegmental planes during surgery and reduce their clarity. In this method, after dividing the bronchus of the segment to be resected, the inflation of the remaining lung may alter the segmental demarcations through collateral pathways, potentially compromising the target segment. To overcome this limitation, Kamiyoshihara et al. investigated oxygen inflation through the divided distal bronchial stump as a means to ventilate the target segment and ensure safe surgical margins [20]. Additionally, in their segmentectomy case series using the IDM, Dai et al. reported that although the technique is feasible, the prolonged duration and challenges in forming adequate intersegmental planes in emphysematous cases present significant limitations [21]. In recent years, studies have supported the idea that the ICG method under infrared fluorescence is easily applicable and provides clearer visualization of intersegmental planes [12,22]. However, this method may fail in patients with extensive anthracosis or iodine allergy [23]. Currently, there is limited research specifically addressing long-term outcomes, such as locoregional recurrence rates, of methods used for intersegmental plane delineation during pulmonary segmentectomy. Most existing studies have primarily focused on the technical feasibility and immediate safety of these methods. Funai et al. reported a 100% five-year cause-specific survival rate in patients undergoing segmental resection with ICG fluorescence imaging. However, the authors noted that the small sample size and single institutional setting of the study limited the generalizability of these findings [24]. Several studies comparing these two methods have focused on achieving clearer identification of intersegmental planes. However, our study differs from others by specifically evaluating margin quality. Preoperatively, the segment containing the nodule and its distance from intersegmental planes were determined using tomography and 3D reconstructions. Our research aimed to identify which method more accurately corresponds to this calculated margin and demonstrated that the ICG method is more effective in achieving higher-quality margins. From an oncological perspective, both methods were found to be successful; however, the IDM exhibited greater deviations from the preoperatively determined margin. One of the primary reasons for this discrepancy was thought to be the difficulty in placing the endostapler along the intersegmental planes defined after lung inflation. These results were as expected in our study. There was no difference between the methods in terms of margin distances. Similarly, Sun et al. showed that both methods clearly revealed the intersegmental plan and that the plans determined by the two methods were completely compatible [10]. The difference in our study is the comparison with the margins determined by 3D modeling. In terms of margin quality, results closer to virtual margins were obtained with the ICG method. Although the IDM was successful in terms of compliance with oncologic principles, we concluded that there may be a negative effect on pulmonary function. There are no specific studies evaluating the long-term effects of both methods on pulmonary functional capacity. Therefore, prospective, randomized controlled trials examining the effects of both methods on pulmonary functional capacity are needed. Although the overall complication rate was low in our study, prolonged postoperative air leakage was observed exclusively in patients who underwent segmentectomy using the IDM. Similarly, Tao et al. reported prolonged air leakage as the most common complication in their study utilizing the IDM [25]. Additionally, other potential complications include positive tumor margins, air leakage due to inadequate endostapler closure, incomplete resection, atelectasis and infection resulting from incorrect vascular division, and unnecessary lung parenchyma removal due to poorly defined margins [26,27].

This study has several limitations. First, it was designed as a retrospective study. As with all retrospective studies, there is a potential for selection bias. Second, the sample size was not sufficiently large, and a prospective study with a larger case series would be necessary for more robust analyses. Third, due to the single-center design, the generalizability of the findings is limited. Additionally, this study primarily focuses on perioperative outcomes, and long-term oncological and functional results were not assessed. Future studies with extended follow-up are needed to evaluate long-term survival and recurrence rates.

## 5. Conclusions

In conclusion, our study demonstrated that the ICG method is superior to the IDM in determining margin quality. As segmentectomy is becoming an inevitable standard treatment for early-stage lung cancer, the widespread adoption of preoperative 3D modeling and intraoperative ICG guidance under near-infrared fluorescence is essential to ensure its safe implementation with minimally invasive surgery and adherence to oncological principles. Nonetheless, while early results are promising, more comprehensive, multicenter studies are needed to definitively determine long-term oncologic outcomes, including locoregional recurrence rates associated with ICG-guided intersegmental plane delineation during pulmonary segmentectomy.

## Figures and Tables

**Table 1:** Distribution of patient demographics and operative results according to groups.

Variables	Total (n = 63)	Group 1 (n = 42)	Group 2 (n = 21)	*p*
Age (year, mean ± SD)	60.03 ± 12.63	60.19 ± 13.66	59.71 ± 10.55	0.73
Sex [n (%)]				0.17
Male	36 (57.1)	27 (64.3)	9 (42.9)	
Female	27 (42.9)	15 (35.7)	12 (57.1)	
Nodule Size (mm, mean ± SD)	14.71 ± 8.09	15.5 ± 9.19	13.14 ± 5.09	0.53
Nodule Type [n (%)]				0.63
GGO	14 (22.2)	9 (21.4)	5 (23.8)	
Semi-solid	8 (12.7)	4 (9.5)	4 (19.0)	
Solid	41 (65.1)	29 (69.1)	12 (57.1)	
Nodule Follow-up [month, median (IQR)]	7 (13)	6 (10)	12 (12)	0.05
Histology [n (%)]				0.49
Primary	46 (73)	29 (69.1)	17 (81.0)	
Secondary	6 (9.5)	4 (9.5)	2 (9.5)	
Other	11 (17.5)	9 (21.4)	2 (9.5)	
Segmentectomy Type [n (%)]				0.15
Simple	21 (33.3)	17 (40.5)	4 (19)	
Complex	42 (66.7)	25 (59.5)	17 (81)	
Segmentectomy Side [n (%)]				
Right	38 (60.3)	25 (59.5)	13 (61.9)	-
Left	25 (39.7)	17 (40.5)	8 (38.1)	
Upper Lobe	38 (60.3)	25 (59.5)	13 (61.9)	-
Lower Lobe	25 (39.7)	17 (40.5)	8 (38.1)	
Segmentectomy [n (%)]				-
S1	13 (20.6)	9 (21.4)	4 (19)	
S2	6 (9.5)	3 (7.1)	3 (14.3)	
S1+2	6 (9.5)	4 (9.5)	2 (9.5)	
S3	5 (7.9)	4 (9.5)	1 (4.8)	
S2+3	1 (1.6)	0	1 (4.8)	
S1+2+3	4 (6.3)	3 (7.1)	1 (4.8)	
S4+5	3 (4.8)	2 (4.8)	1 (4.8)	
S3+4+5	1 (1.6)	1 (2.4)	0	
S6	14 (22.2)	12 (28.6)	2 (9.5)	
S7	2 (3.2)	0	2 (9.5)	
S7+8	1 (1.6)	1 (2.4)	0	
S10	2 (3.2)	1 (2.4)	1 (4.8)	
S6+10	1 (1.6)	1 (2.4)	0	
S9+10	3 (4.8)	0	3 (14.3)	
S8+9+10	1 (1.6)	1 (2.4)	0	
Introperative Complication [n (%)]				0.54
No	61 (96.8)	40 (95.2)	21 (100)	
Yes	2 (3.2)	2 (4.8)	0	
Postoperative Complication [n (%)				0.16
No	57 (90.5)	36 (85.7)	21 (100)	
Yes	6 (9.5)	6 (14.3)	0	
Chest Tube Removal [day, median (IQR)]	2 (1)	2 (1)	2 (1)	0.43
Postoperative LOS [day, median (IQR)]	3 (2)	3 (1.3)	3 (1)	0.05

(GGO: ground glass opasity, IQR: interquartile range, LOS: length of hospital stay, mm: millimeter, SD: standard deviation).

**Table 2:** Comparison of intersegmental margin measurements and margin quality between groups.

Variables	Total (n = 63)	Group 1 (n = 42)	Group 2 (n = 21)	*p*
Virtual Margin (mm, mean ± SD)	21.52 ± 7.07	21.14 ± 6.44	22.28 ± 8.30	0.92
Pathological Margin (mm, mean ± SD)	15.15 ± 9.48	14.14 ± 9.23	17.19 ± 9.87	0.21
Margin Deviation (mm, mean ± SD)	7.25 ± 5.16	8.28 ± 5.52	5.19 ± 3.64	0.04
Margin Success [n (%)]				0.05
Yes	46 (73)	27 (64.3)	19 (90.5)	
No	17 (27)	15 (35.7)	2 (9.5)	

(mm: millimeter, SD: standard deviation).

## Data Availability

The original contributions presented in this study are included in this article material. Further inquiries can be directed to the corresponding author.
